# TGFß1 Stimulates the Over-Production of White Matter Astrocytes from Precursors of the “Brain Marrow” in a Rodent Model of Neonatal Encephalopathy

**DOI:** 10.1371/journal.pone.0009567

**Published:** 2010-03-05

**Authors:** Jennifer M. Bain, Amber Ziegler, Zhengang Yang, Steven W. Levison, Ellora Sen

**Affiliations:** 1 Department of Neurology and Neurosciences, UH Cancer Center and the Graduate School of Biomedical Sciences, New Jersey Medical School, University of Medicine and Dentistry of New Jersey, Newark, New Jersey, United States of America; 2 National Brain Research Center, Manesar, Haryana, India; 3 Institute of Brain Science and State Key Laboratory of Medical Neurobiology, Fudan University, Shanghai, People's Republic of China; The Mental Health Research Institute of Victoria, Australia

## Abstract

**Background:**

In children born prematurely and those surviving cerebral ischemia there are white matter abnormalities that correlate with neurological dysfunction. Since this injury occurs in the immature brain, when the majority of subventricular zone (SVZ) cells generate white matter oligodendrocytes, we sought to study the effect this injury has on gliogenesis from the SVZ. We hypothesized that there is aberrant glial cell generation from the SVZ after neonatal hypoxia ischemia (H/I) that contributes to an increased astrogliogenesis with concomitant oligodendroglial insufficiency. Mechanistically we hypothesized that an increase in specific locally produced cytokines during recovery from injury were modifying the differentiation of glial progenitors towards astrocytes at the expense of the more developmentally-appropriate oligodendrocytes.

**Methodology/Principal Finding:**

For these studies we used the Vannucci H/I rat model where P6 rats are subjected to unilateral common carotid ligation followed by 75 min of systemic hypoxia. Retroviral lineage tracing studies combined with morphological and immunohistochemical analyses revealed the preferential generation of SVZ-derived white matter astrocytes instead of oligodendrocytes post hypoxia/ischemia. Microarray and QRT-PCR analyses of the damaged SVZ showed increased expression of several cytokines and receptors that are known to promote astrocyte differentiation, such as EGF, LIF and TGFß signaling components. Using gliospheres to model the neonatal SVZ, we evaluated the effects of these cytokines on signal transduction pathways regulating astrocyte generation, proliferation and differentiation. These studies demonstrated that combinations of EGF, LIF and TGFß1 reconstituted the increased astrogliogenesis. TGFß1-induced Smad 2/3 phosphorylation and the combination of EGF, LIF and TGFß1 synergistically increased STAT3 phosphorylation over single or double cytokine combinations. Pharmacologically inhibiting ALK5 signaling *in vitro* antagonized the TGFß1-induced increase in astrocyte generation and antagonizing ALK5 signaling *in vivo* similarly inhibited astrogliogenesis within the SVZ during recovery from H/I.

**Conclusion/Significance:**

Altogether, these data indicate that there is aberrant specification of glial precursors within the neonatal SVZ during recovery from neonatal H/I that is a consequence of altered cytokine signaling. Our studies further suggest that antagonizing the ALK5 receptor will restore the normal pattern of cell differentiation after injury to the immature brain.

## Introduction

Neonatal hypoxia/ischemia (H/I) is a major cause of morbidity resulting from complications during the birthing process that leads to cognitive, sensory and motor deficits in approximately 25% of affected infants. Accumulating evidence has demonstrated that immature oligodendrocytes are extremely sensitive to hypoxia, oxidative stress and glutamate, which are thought to contribute to the focal cystic degeneration as well as to diffuse injury seen in the periventricular white matter. Investigators are also beginning to appreciate the impact of this injury on the SVZ, which is the region of the immature brain that harbors multipotential neural stem/progenitors, endowed with the ability to regenerate neurons, astrocytes and oligodendrocytes. Using the classic Vannucci H/I animal model [1], [2], [3], [4], we have previously demonstrated that neonatal white matter immature oligodendrocytes undergo acute excitotoxic cell death as do progenitors in the SVZ following H/I, while the stem cells are resilient to death effectors [5], [6], [7], [8]. Not only are the stem cells resilient, but several studies have shown that they expand subsequent to H/I and can regenerate neurons and possibly some oligodendrocytes [9], [10], [11], [12], [13]. Since the stem cells in the SVZ are resilient to H/I and in fact participate in CNS regeneration, the failure of the white matter to regenerate is not easily reconciled.

Studies characterizing the cells in the neonatal SVZ have demonstrated that in addition to the tripotential stem/progenitors there are more restricted bipotential glial progenitors (BGP) that are capable of producing oligodendrocytes or astrocytes developmentally [14], [15], [16], [17], [18], [19]. The decision to produce one cell type versus the other appears to be dictated by signals that the progenitors receive from their local environment [20]. In the adult brain, mature resting astrocytes can be induced to become reactive, a process known as reactive gliosis, as a consequence of cytokines produced by damaged neurons and reactive microglia. Histopathologic studies on the brains of infants who have expired after neonatal H/I have shown similar gliosis in affected regions [21], [22]. However, astrocyte generation in the neonatal brain is incomplete at the time of injury, thus the reactive astrocytes in the neonatal brain may have a different origin than those in the adult brain. Accordingly, we hypothesized that the gliosis in the infant brain and the failure of oligodendrocyte regeneration after neonatal H/I may be linked and that the extrinsic signals that determine sequential cell fate specification within the postnatal SVZ during normal development are disturbed following H/I thus shifting the production in the SVZ from oligodendrocytes to astrocytes. This mis-specification will then contribute to the permanent deficit in periventricular white matter oligodendrocytes subsequent to H/I. The objective of this study was to test this hypothesis and to identify the specific signals that coordinate the differentiation of the glial progenitors of the SVZ.

## Results

### Aberrant Glial Specification from the SVZ after Neonatal H/I

**Figure 1:**
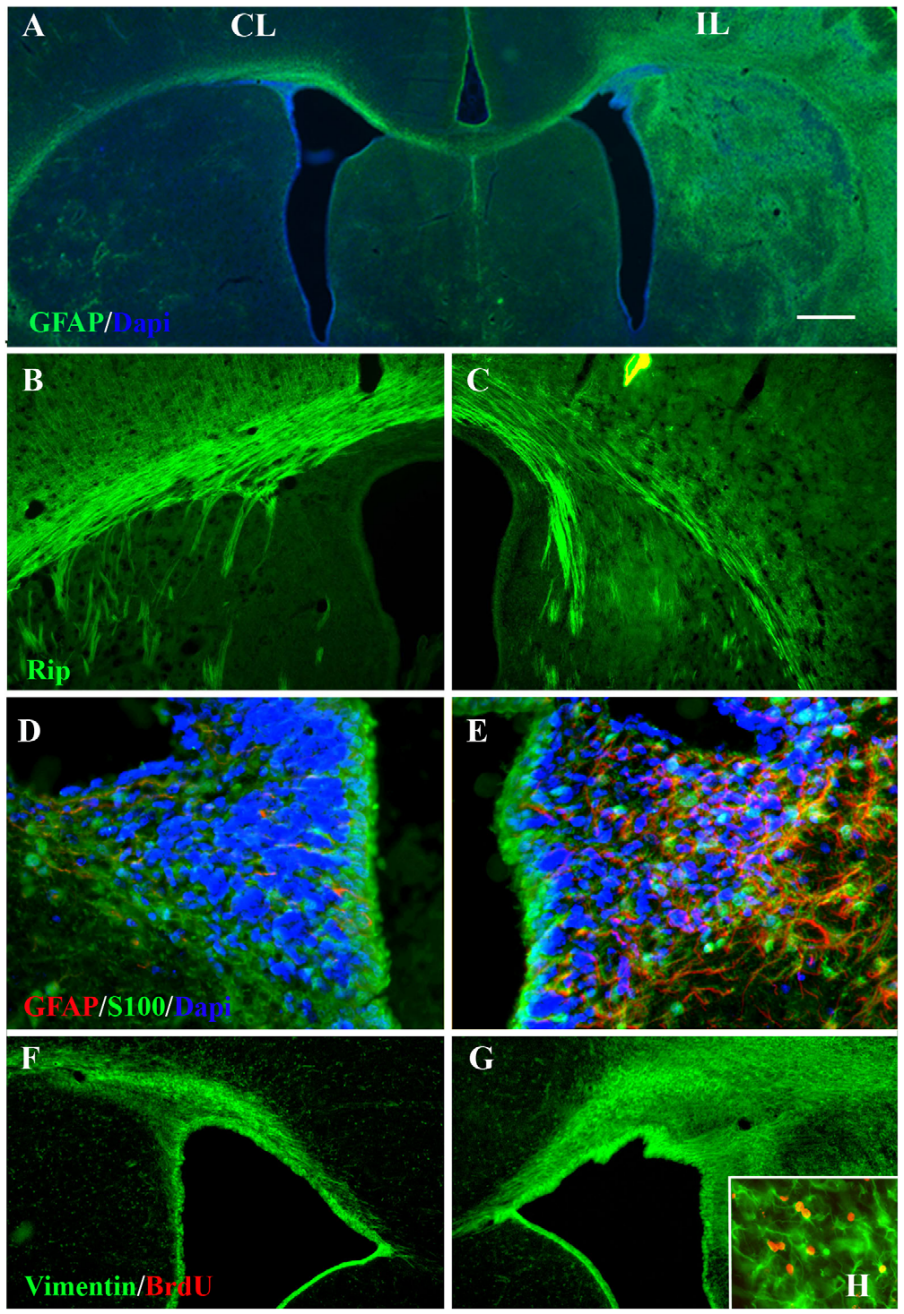
Over-production of astrocytes in the injured hemisphere at 7 days of recovery as a consequence of neonatal H/I. (**A**) Sections from animals sacrificed at 7 days of recovery were stained with antibodies against GFAP (green) and counterstained with DAPI (blue). (**B,C**) Sections were stained with Rip for oligodendrocytes and myelin in the ILH (C) compared to the CLH (B). (**D,E**) Sections were stained with antibodies against GFAP (red), S100b (green) and counterstained with DAPI (blue) (E) depicts the ILH; (D) depicts the CLH (D). (**F,G**) The ILH SVZ (G) was stained with antibodies against vimentin (green); (F) depicts the CLH. (**H**) Inset depicts BrdU+/Vim+ cells in the ILH SVZ from animals injected with BrdU (red) at day 3 of recovery and analyzed at 7 days of recovery. Scale bar represents 100 µm (A), 50 µm (B,C,F,G), 25 µm (D,E,H).

We evaluated gliogenesis subsequent to H/I using immunofluorescence methods as well as by evaluating cell proliferation within the SVZ and by fate mapping SVZ cells using replication deficient retroviruses. Using the Rip antibody to visualize immature and mature oligodendrocytes we observed significantly less myelin and fewer oligodendrocytes in the ipsilateral hemisphere (ILH) (Fig. 1C) compared to the contralateral hemisphere (CLH) at 7 days of recovery (Fig. 1B). This decrease in myelin staining was accompanied by a dramatic increase in the number of GFAP positive cells (Fig. 1A). At 7 days of recovery, significant increases in immature astrocytes were observed in the ILH, as assessed by increased vimentin, S100b and GFAP staining (Fig. 1E,G) compared to the CLH (Fig. 1D,F). Many of these astrocytes were newly born cells, as there was an increase in stellate BrdU+/Vimentin+ cells around the lateral ventricles at 7 days recovery post H/I (Fig. 1H). These observations are consistent with the hypothesis that H/I decreases the overall population of mature myelinating oligodendrocytes and induces astrocyte production.

**Figure 2:**
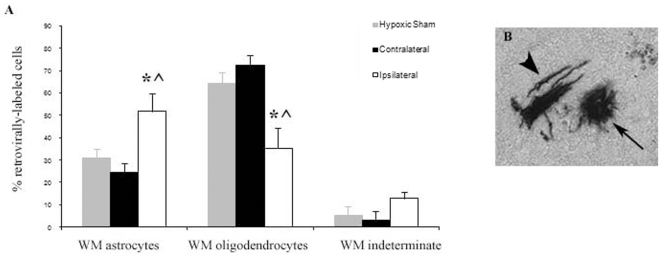
SVZ cells preferentially generate white matter astrocytes rather than oligodendrocytes after H/I. Replication-incompetent retroviruses containing the alkaline phosphatase (DAP) reporter gene were stereotactically injected at P8 into the lateral ventricles (two days after H/I). For quantitative analysis at 10 days after DAP injection (A) cell types within the white matter (WM) were classified based upon morphological criteria as either astrocytes (arrow), oligodendrocytes (arrowhead) or indeterminate (B). Data are averaged from 8 hypoxic sham, 8 contralateral hemispheres (CL), and 7 ipsilateral hemispheres (IL) * indicates p<0.05 compared to CLH, ∧ indicates p<0.05 compared to hypoxic sham animals as determined by ANOVA with Fisher's PLSD post-hoc test.

To determine whether there was a shift in glial specification in the periventricular white matter from cells with origins in the SVZ, we performed replication-deficient retrovirus fate-mapping. Retroviruses were stereotactically injected bilaterally into the lateral ventricles 2 days after neonatal H/I. These retroviruses label mitotically active cells, including SVZ progenitors. Indeed at 2 days of recovery almost all of the retrovirally labeled cells were located within the SVZ (Fig. S1C,D). By 12 days of recovery from H/I, most of the infected cells had migrated out of the SVZ and were mostly located in the white matter of the corpus callosum (Fig. S1B). A morphological analysis of glial phenotypes revealed almost a doubling in the percentage of astrocytes with a concurrent 50% decrease in both the percentage of myelinating oligodendrocytes and total oligodendrocytes (Fig. 2A, B; p<0.05).

**Figure 3:**
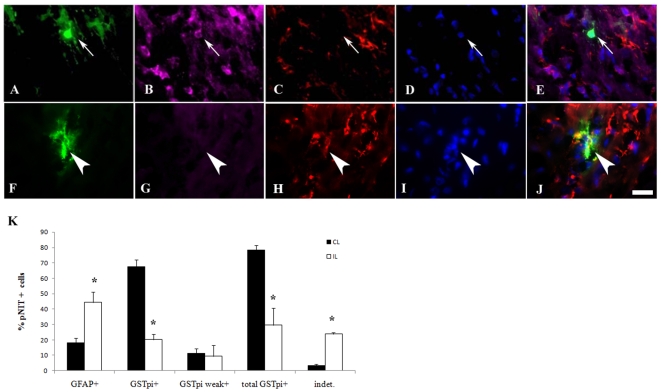
SVZ cells preferentially generate GFAP+ astrocytes in white matter instead of GST π + oligodendrocytes. Two µL of pNIT replication-incompetent retroviruses containing the GFP reporter gene were stereotactically injected at P8 into the lateral ventricles (two days after H/I). Cryostat sections (15 µm) were prepared after sacrifice at P27. Sections were stained for GFP (A,F), GST π (B,G) and GFAP (C,H) followed by DAPI counterstain (D,I). Merged color overlays are presented in panels E and J. Cell types within the corpus callosum (WM) were counted as GFAP +, GSTπ +, GSTπ weakly + and double negative (indeterminate). Panels A–E illustrate a representative GFAP+ cell, and panels F–J illustrate a GSTπ+ cell. Panel K depicts quantitative data averaged from 3 IL hemispheres and 6 CL hemispheres. * indicates p<0.05 compared to CLH as determined by student's t-test. Scale bar represents 20 µm.

We also used a GFP encoding retrovirus together with immunofluorescence for astrocytic Glial Fibrillary Acidic Protein (GFAP) and oligodendrocytic Glutathione-s-Transferase pi (GSTπ). For these studies, we used a lower titer of retrovirus and used DAPI to identify individual cells prior to classifying them as GFAP+, GSTπ+, weakly GSTπ+, or double negative (indeterminate) (Fig. 3A,B). Cells were evaluated at 3 weeks after injury to enable them to more fully mature. At this time point, majority of labeled cells were in the white matter. Confirming the data provided in Fig. 2, which were collected at 12 days after injury, the percentage of GFAP+ astrocytes increased from 18% to 45%, with a concomitant decrease in the percentage of GSTπ+ oligodendrocytes from almost 80% in the ILH to 30% in the CLH (Fig. 3C, p<0.05). We also found an increase in the percentage of indeterminate cell types in the ILH (increase from 3% to 24%, p<0.05). Moreover, there was a small population cells with the morphology of oligodendrocytes, that only weakly stained positive for GSTπ, and these cells also increased in the ILH compared to the CLH.

Reproducibly, in both the DAP and pNIT retrovirus experiments, there were significantly fewer labeled cells in the ILH (Fig. S1) as compared to the CLH. To determine whether this was due to fewer cells being infected 2 days after injury when the viruses were injected, we injected 5 uL of pNIT 2 days after H/I and the animals sacrificed 48 h later. pNIT labeled cells were predominantly located in the SVZ, however, some cells had migrated into the subcortical white matter. Echoing the results from the 2 and 3 week survival time points, at this earlier time point we observed that there were few infected cells in the ILH as compared to the CLH (Fig. S1A,C,D).

### Signals Known to Induce Astrocytic Differentiation Are Elevated in the Ipsilateral SVZ 7 Days after Injury

We hypothesized that extrinsic factors were responsible for the aberrant production of astrocytes from the SVZ after H/I. Therefore, to establish which genes were aberrantly expressed we performed a gene array analysis with QRT-PCR validation. We microdissected the SVZ and compared the RNA profiles from ILH to the CLH using a commercially available nylon array. A total of 11 genes were changed at least 2 fold with very few genes showing decreased expression at 7 days of recovery from H/I (data not shown). Of the genes whose expression were elevated in the ipsilateral SVZ after injury on an average by at least 2-fold in three independent experiments, 3 families caught our attention that have previously been implicated in astrocyte specification; EGF, LIF and TGFß. QPCR revealed no change in EGF mRNA expression at 7 days of recovery from H/I between ILH and CLH [23]. However, consistent with the array results, there was a 2-fold increase in LIF and a 4-fold increase in TGFß1 (Table 1). While there was no change in the expression of LIFR, the expression of gp130 and EGFR were elevated by 1.5- and 1.44-fold, respectively. The mRNA levels for the TGFβ1 receptor, ALK1 was decreased by 0.6-fold, whereas levels for the ALK5 receptor increased 1.8-fold. Other growth factors, including Notch, FGF and BMP family members and their corresponding receptors, as well as the cell surface glycoprotein CD44 were also increased on the SuperArray, however, changes in their expression levels remain to be validated (Table S1).

**Table 1:** Quantitative PCR analysis for genes associated with astrogliogenesis following neonatal H/I.

Gene	Fold Change at 7 days of recovery
EGFR	1.44
LIF	2.1
LIFR	1.1
gp130	1.5
TGFβ1	4.1
ALK1	0.625
ALK5	1.8

Ipsilateral and contralateral SVZs were dissected at 7 d post H/I. Total RNA was isolated and amplified by Q-PCR using Taqman primers specific for the genes shown in the table, and normalized to the expression of 18S RNA. Values for all transcripts except LIFR were significantly different from the CLH as determined by the REST program.

### Effects of EGF, LIF and TGFß1 on Astrocyte Production from Gliospheres

**Figure 4:**
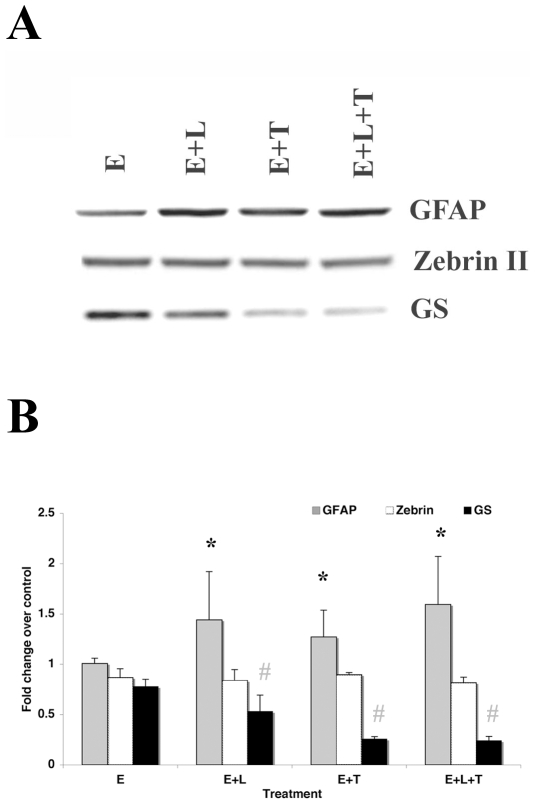
Combinations of EGF, LIF and TGFß1 effectively increase astrocyte generation in gliosphere cells. Gliospheres were grown in ProN supplemented with B104 conditioned medium and 10 ng/ml FGF-2. They were dissociated and plated onto culture dishes for 6 h in differentiation media with 2% serum and then switched to differentiation media supplemented with either 5 ng/mL EGF (E), LIF (L), TGFß1 (T) or combinations for 48 h. (**A**) Densitometric analyses of protein levels on Western Blots were performed for GFAP, Zebrin II and GS as indices of astrocyte differentiation. (**B**) Values represent the mean fold change over controls ± SEM from 3 independent experiments. * and # denote a significant increase and decrease respectively from control (P<0.05).

Results from the SuperArray and QT-PCR indicated that there were increases in astrogliogenic cytokines or their receptors in the injured SVZ. To determine whether EGF, LIF and TGFß1 either alone or in combination could promote astrocyte generation, we tested their effects using an *in vitro* model for the glial progenitors of the neonatal SVZ; gliospheres. Gliospheres were treated with physiologically relevant concentrations of EGF, LIF and TGFß1 either alone or in combination and Western blots were performed for GFAP, Zebrin II and glutamine synthetase (GS) as indices of astrocyte generation. No single factor alone significantly altered GFAP expression (data not shown), although GFAP expression was elevated in spheres treated with EGF in combination with LIF and/or TGFß1 (Fig. 4A,B). The combination of EGF, LIF and TGFß1 yielded the highest GFAP expression. The expression of Zebrin II was not significantly affected by treatment with EGF, LIF and TGFß1 either alone or in combinations. A significant decrease in GS expression was observed in cells treated with EGF in the presence of LIF, TGFß1 or a combination of both, as compared to cells treated with EGF alone (Fig. 4A,B).

### Specific Cytokine Combinations Differentially Regulate Expression and Phosphorylation Status of Transcription Factors

**Figure 5:**
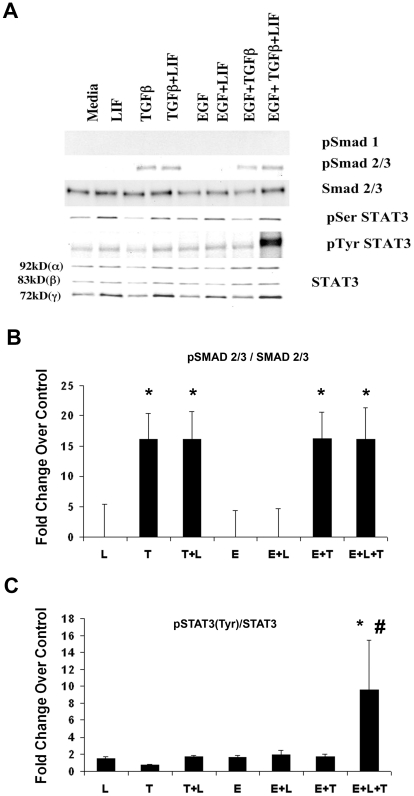
Differential effect of EGF, LIF and TGFß1 on Smad 2/3 and STAT3 phosphorylation in gliospheres. Gliospheres were grown in ProN supplemented with B104 conditioned medium and 10 ng/ml FGF-2. They were dissociated and plated onto culture dishes and then treated with 5 ng/mL EGF (E), LIF (L), TGFß1 (T) either alone or combinations in differentiation media for 30 min. (**A**) Total cell lysates were collected and Western blot analysis was performed to determine levels of total Smad2/3, STAT3, pSmad 2/3 on Ser465/467 and pSTAT3 on Ser727 and Tyr705 residues. (**B**) Values represent fold change in pSmad2/3 over Smad2/3. (**C**) Values represent fold change in pSTAT3^Tyr705^ over STAT3, ± SEM from 3 independent experiments. * Denote significant increase from control, # significant increase from treatment with a single cytokine or combination of two (P<0.05).

As combinations of cytokines were more effective than single factors we hypothesized that they were synergistically activating intracellular signaling pathways. We analyzed the status of STAT and Smad transcription factors, which are known to be associated with astrogliogenesis. When tested alone, EGF had little effect on STAT and Smad phosphorylation. LIF increased the phosphorylation of STAT-3 on both serine 727 and tyrosine 705, both in the presence and absence of TGFß1 or EGF. While treatment with neither LIF nor EGF affected pSmad2/3 levels, TGFß1 induced Smad 2/3 phosphorylation. This heightened pSmad2/3 level observed in TGFβ-treated cells was unaffected by the presence of either EGF, LIF or a combination of both LIF and TGFβ (Figure 5A,B). Phosphorylation of Smad1 was undetectable under all conditions tested (Fig. 5A). The levels of total STAT3, pSTAT3^Ser^ and pSTAT3^Tyr^ were elevated in the presence of LIF (Fig. 5A). The elevated levels of pSTAT3^Ser^ were comparable in cells treated with LIF either in the presence or absence of EGF or TGFβ or both. However, an approximately 9 fold increase in pSTAT3^Tyr^ level was observed in cells treated with a combination EGF, LIF and TGFβ1, as compared to those treated with LIF alone or with EGF or TGFβ1 (Fig. 5A,C).

### TGFß1 Synergizes with EGF to Induce Astrocyte Proliferation and Antagonizing ALK5 Blocks This Astrocyte Generation *In Vitro*

**Figure 6:**
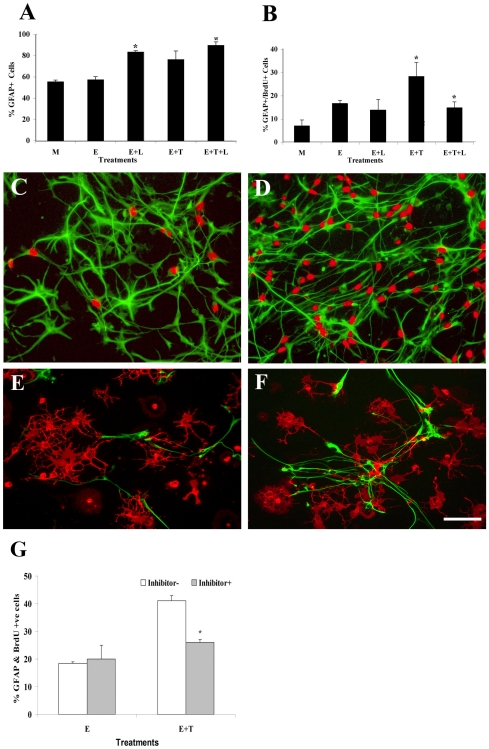
TGFß1 induced increased astrocyte proliferation is mediated through the ALK5 receptor. Gliospheres were plated onto chamber slides in differentiation media supplemented with 5 ng/mL of EGF (E), LIF (L), and TGFß1 (T) in different combinations for 96 h and were pulsed with BrdU for the last 3 h in culture. The percentage of (**A**) GFAP+ and (**B**) GFAP+/BrdU+ cells is depicted. (**C,D**) Representative images of gliospheres differentiated in EGF (C) or combination of EGF and TGFß1 (D) stained for GFAP (green) and red (BrdU). (**E,F**) Gliospheres were similarly treated with EGF or a combination of EGF and TGFß1 for 96 hrs and the effect of these cytokines on oligodendrocyte generation were determined by O4 staining. Representative images of gliospheres differentiated in EGF (E) or combination of EGF and TGFß1 (F) stained for GFAP (green) and red (O4) are shown. (**G**) Gliospheres were propagated in EGF or EGF+ TGFß1 in the presence or absence of 10 µM SB431542, a potent ALK5 inhibitor, pulsed with BrdU and stained for GFAP and GFAP/BrdU (G). Results are representative of 3 independent experiments. * indicate statistically significant differences (p<0.05) for each condition compared to medium alone (M) controls using Student's t-test. The scale bar represents 35 µm for panels C,D and 50 µm for panels E,F.

The changes we observed in the levels of astrocytic proteins could occur either as a result of increased astrocyte numbers or increased expression of astrocyte markers. To distinguish between these two possibilities, we dissociated the gliospheres, stimulated the single cells with cytokines and counted the percentage of astrocytes by GFAP immunostaining. We found that no factor alone increased the percentage of astrocytes. When these factors were combined, LIF together with EGF significantly increased the percentage of GFAP positive cells (Fig. 6A). However, it was the combination of all three cytokines that most significantly increased astrocyte numbers compared to controls (Fig. 6A).

In order to determine whether cell proliferation was responsible for this increase in astrocytes, gliospheres were treated with EGF in combination with LIF and TGFß1 followed by a short BrdU pulse. We found that the combination of EGF with TGFß1 stimulated the incorporation of BrdU into the GFAP+ cell population (Fig. 6B,C,D). Interestingly, LIF suppressed the proliferative effects of EGF and TGFß1, by decreasing the number of BrdU+/GFAP+ cells (Fig. 6B). We also observed that specific cytokines altered the morphology of GFAP+ cells, as gliosphere cells cultured in the presence of EGF produced GFAP+ cells with broad, flattened processes whereas the addition of TGFß1 produced cells with highly stellate, thin processes (Fig. 6C,D). While EGF and TGFβ1 increased astrocyte proliferation, growth in EGF either alone or in combination with TGFβ1 had no effect on oligodendrocyte progenitor production. The number of O4 positive cells obtained in EGF alone or in combination with TGFβ1 was comparable (Fig. 6E, F).

As TGFß1 induced SVZ-derived gliospheres to produce more astrocytes and the signal transduction studies indicated that TGFß1 was stimulating the ALK5 receptor, we hypothesized that inhibiting the ALK5 receptor would inhibit astrocyte generation. Treatment with ALK5 antagonist SB431542 had no effect on BrdU incorporation into gliosphere cells stimulated with EGF alone. However, as expected SB431542 completely inhibited the EGF and TGFβ1–stimulated increase in proliferating astrocytes as measured by BrdU incorporation (Fig. 6G).

### Increased Astrogliosis after Hypoxia/Ischemia Is Abrogated by ALK5 Inhibition *In Vivo*

**Figure 7:**
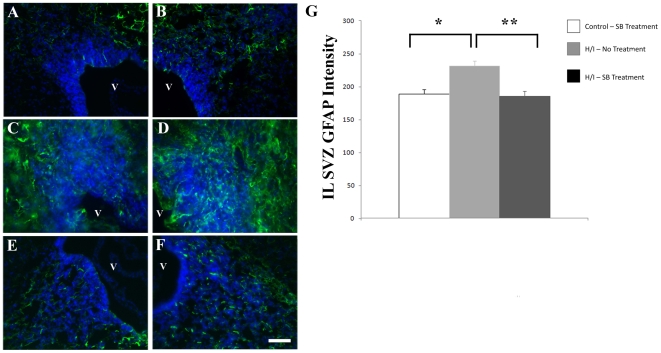
Increased astrogliosis at 4 days of recovery observed in the injured SVZ after H/I is decreased by ALK5 inhibition *in vivo*. (**A,B**) Representative images of CL (A) and IL (B) hemispheres from age-matched control animals (twice daily i.p. SB505124 injections) and stained with antibodies against GFAP (green) and counterstained with DAPI (blue). (**C,D**) Images of CL (C) and IL hemispheres (D) of H/I vehicle-treated animals. (**E,F**) Representative images of CL (E) and IL (F) of SB505124-treated animal at 4 days after injury. (**G**) Densitometric analyses of GFAP intensity within the SVZ in H/I and H/I + SB treated animals at 4 days after injury. Untouched controls treated with SB compound were also analyzed and no significance was found between IL and CL hemisphere data (data not shown). * and ** denote p<0.05 using ANOVA analysis with Fisher's PLSD post-hoc test comparing vehicle to H/I and H/I to H/I + SB respectively. Scale bar represents 50 µm.

Based on the findings above that inhibiting ALK5 diminished astrocyte generation *in vitro*, we tested the hypothesis that inhibiting ALK5 after neonatal H/I would similarly reduce astrogliosis *in vivo*. At 4 days after H/I, astrogliosis in the injured SVZ was observably higher (Fig. 7C,D) than in the contralateral SVZ (Fig. 7A,B) as shown by intense GFAP expression. To antagonize ALK5 *in vivo*, we administered SB505124 to animals immediately after H/I, and twice daily for 4 days after injury and analyzed GFAP intensity in each hemisphere at 4 days of recovery. Unlike SB431542, this ALK5 kinase inhibitor crosses the blood brain barrier [24], [25]. Visually, there was a clear decrease in staining for GFAP (Fig. 6E,F), and a quantitative analysis of fluorescence intensity confirmed that SB505124 treatment significantly decreased GFAP expression in the damaged SVZ (Fig. 7G, p<0.05). Although SB505124 prevented the increase in GFAP staining within the SVZ, GFAP staining intensity in the un-injured hemisphere was not different between vehicle-and SB505124 treated animals, and the injury-induced increase in GFAP was indistinguishable in neocortex of vehicle and SB505124 treated animals (Figure S2). Thus, administering an ALK5 antagonist *in vivo* appears to abrogate the injury-induced astrocyte generation in the SVZ. At this early time-point of recovery, we did not observe any change in staining for the oligodendrocyte marker Rip within the subcortical white matter or SVZ (Data not shown [26]).

## Discussion

Neonatal white matter damage occurs during that interval of human development when SVZ cells are predominantly producing oligodendrocytes. This injury occurs when the late OPCs are most abundant [27], [28], [29] and studies show that these progenitors are extremely vulnerable to glutamate, oxidative stress and FasL [30], [31], [32], [33], [34], [35], [36]. Recent studies indicate that oligodendrocyte progenitors are present within abnormal white matter of human PVL cases, but are unable to properly differentiate and to replace damaged OPCs [37], [38]. Back et al (2005) suggested that certain factors made by reactive astrocytes, such as hyaluronan, accumulate in white matter lesions that inhibit the maturation of oligodendrocyte progenitors both in human multiple sclerosis lesions and in animal models of experimental autoimmune encephalitis (EAE) [39]. Thus reactive gliosis subsequent to neonatal white matter injury may directly contribute to the failure of myelin formation.

Our studies and others have established that there is an expansion of the SVZ neural stem/progenitor cell population, resulting in neuronal and some glial cell replacement [7], [9], [10], [11], [13], [40]. Therefore, this regenerative response should lead to significant OPC replacement, especially since this injury is occurring precisely when the majority of OPCs are being generated. However, OPC replacement in both rat models and in human infants is limited. We hypothesized that the signals that determine sequential cell fate specification within the SVZ are disturbed following H/I adversely affecting gliogenesis in the developing brain. In the studies reported here we analyzed (1) whether there is aberrant generation of oligodendrocytes and astrocytes from SVZ progenitors subsequent to neonatal H/I; (2) whether there is increased expression of extrinsic signals that promote astrocyte differentiation in the SVZ following H/I; (3) whether those signals that are aberrantly expressed promote astrocyte differentiation from glial restricted progenitors and (4) whether inhibiting one of those signals would prevent aberrant astrogliogenesis.

We observed increased astrogliosis in the injured hemisphere at 4 and 7 days of recovery. This end result could have occurred though 3 possible routes: 1) OPC death, 2) astrocyte proliferation or 3) aberrant differentiation of SVZ glial progenitors into astrocytes. Were only scenario 1 occurring, then one would predict that the regenerative expansion of SVZ precursors would replace the depleted oligodendrocyte progenitors, which is incomplete at best. Scenario 2 is surely occurring as numerous studies have shown local astrocyte proliferation after H/I, however; to our knowledge none attribute this astrogliosis to aberrant production of astrocytes from the glioblasts in the SVZ [41], [42], [43], [44], [45], [46], [47], [48], [49]. Using retrovirus fate mapping techniques and immunohistochemistry for lineage-specific markers, we provide data that strongly support the third scenario - that SVZ glial progenitors are aberrantly differentiating after injury. Importantly, we show that some of the new astrocytes that are produced are derived from SVZ cells as opposed to locally proliferating immature astrocytes confirming our hypothesis that gliosis in the immature brain may be qualitatively different from gliosis in the adult brain. In this manuscript, we propose an alternative mechanism for the generation of “excess” astrocytes in the injured brain–altered generation of astrocytes from subventricular zone progenitors. This alternative source may be further amplified by astrocyte proliferation locally.

Interestingly, we also observed an increase in the percentage of weakly stained GSTπ+ cells and indeterminate cell types in the injured white matter, which is reminiscent of the halt in the differentiation of OPCs as recently observed in human PVL cases [37], [38]. Our SuperArray results indicate an up-regulation of CD44 in the injured SVZ, the cell surface glycoprotein capable of binding hyaluronic acid, supporting a potential role for hylauronan as an inhibitor of oligodendrocyte production after injury. Additionally, we found that there were significantly fewer labeled cells in the injured hemisphere as compared to the contralateral and control hemispheres. As we have previously demonstrated that there is a depletion of progenitors acutely after neonatal H/I [50], [51], [52] the depletion of these progenitors could reduce the proportion of cells available for retroviral infection. To test this hypothesis, we examined the extent of retrovirus infection 2 days after injury and observed far fewer infected cells in the damaged SVZ and in the corpus callosum 48 h after infection. Thus, the expansion of the stem/progenitor cells in the SVZ that we have previously documented may not be sufficient to replenish damaged white matter oligodendrocytes progenitors. However, we cannot rule out the possibility that SVZ cells that divided after the injury may fail to survive and that the reduced number of retrovirally labeled cells is a consequence of persistent glial progenitor cell death.

Cumulatively, our results demonstrate that there is a shift in the cell types generated from the SVZ, and that the cells that are produced from the SVZ after injury preferentially become astrocytes rather than oligodendrocytes. It is well established that radial glial cells are an important source of astrocytes in the developing brain [53] and recently, Sizonenko et al. 2008 documented accelerated transformation of radial glia into astrocytes as a mechanism for astrogliogenesis after neonatal H/I [54]. Our studies provide an additional mechanism whereby astrocyte generation is enhanced from a different set of precursors, those within the SVZ. It is known that axonal loss will lead oligodendrocyte precursor death [55]. Although axonal loss occurs following neonatal H/I, we have previously shown that this loss is disproportional to decreased oligodendrocyte generation [56]. Therefore, axonal loss is not sufficient to explain the loss of oligodendrocytes, but it may contribute to the decrease in oligodendrocyte replenishment after injury,

We provide evidence both from in vivo analyses as well as from in vitro studies that indicate that elevated levels of cytokines post injury are stimulating astrocyte proliferation. Extrinsic cytokines and growth factors provide decisive signals that govern the differentiation choices of neural precursors [57]. These extracellular signaling molecules work though intracellular signaling cascades to either activate or repress distinct groups of transcription factors. Gene array analysis validated by QRT-PCR revealed an increase in the expression of LIF and TGFß1 as well as their corresponding receptors, LIFR, gp130 and AK5 in the SVZ at 7 days recovery from H/I. We have also found that EGFR is more highly expressed on putative neural stem cells and SVZ progenitors after H/I [23]. These factors have been previously shown to promote astrocyte differentiation from embryonic and fetal neural precursors [58], [59], [60], [61], [62], [63], [64], [65]. As reported here, as well as in a recently published study, we found that there was no change in the expression of EGF in the SVZ after neonatal H/I. This result appears to contradict the importance of EGFR signaling for the astrogliogenesis observed, however, TGFα, not EGF, is the major EGF-R ligand during CNS development where TGFα is expressed 15 to 150 times higher than EGF [66].

To date, the effects of EGF, LIF and TGFß1 have not been fully assessed on SVZ glial progenitors. We found that the combination of EGF with either LIF or TGFß1 increased astrocyte generation from gliospheres, an *in vitro* model for studying SVZ glial progenitors. GFAP expression increased with combinations of these growth factors, and the combination of all 3 was most effective in promoting astrocytic protein expression. Despite increased GFAP expression, astrocyte maturation, as measured by the metabolic marker GS, was considerably reduced in all cultures containing TGFß1. Of relevance to neonatal hypoxia/ischemia, our findings support previous studies showing that TGFß1 impairs astrocyte GS activity [67]. A consequence of reduced GS expression is that the capacity of these astrocytes to detoxify glutamate will be impaired. Without the capacity to eliminate glutamate, OPCs and other cell types will be rendered even more vulnerable to damage and eventual cell death.

Although TGFβ1 has been shown to inhibit astrocyte proliferation [68], [69], it has been shown to increase the proliferation of glioblastoma [70]. As we show here for SVZ glial precursors, these differences in the effects of TGFß1 could be due to varied responsiveness of cells at different stages of differentiation. Previous studies have suggested that proper myelination after CNS injury requires astrocyte participation [71]. Our *in vivo* studies show that astrocytes are being generated from the SVZ after injury, suggesting that these SVZ-derived astrocytes may be proliferating but not maturing properly to actively participate in normal myelination. We found that EGF and TGFß1 were most effective in increasing astrocyte proliferation. These findings indicate that combinations of growth factors promote the proliferation of SVZ-derived immature astrocytes, while retarding their differentiation and that TGFß1 is a key stimulus.

Bone morphogenetic proteins of the TGFß family and LIF have been shown to synergistically induce astrocyte differentiation from fetal neuroepithelial precursors though a complex comprised of the transcription factors Smad1 (via ALK1) and STAT3 respectively, together with the transcriptional co-activator p300 [72]. Analogously, we found that EGF, LIF and TGFß1 promoted astrocyte differentiation from postnatal SVZ glial progenitors and that they synergistically stimulated STAT-3 phosphorylation. These *in vitro* experiments confirm results from an earlier study that showed increased STAT-3 phosphorylation in the injured SVZ after H/I [73]. We hypothesized that TGFß1 would act though ALK1 to induce astrocyte specification; however, TGFß1 only increased Smad 2/3 phosphorylation, reflecting signaling though ALK5 instead of ALK1. This result was not necessarily predictable since ALK1 is activated by BMPs, and has been previously implicated in astrocyte development. Supporting the importance of the ALK5 receptor, pharmacologically inhibiting ALK5 signaling with the potent inhibitor SB431542 [74], [75] prevented TGFβ1-induced immature astrocyte proliferation.

As our *in vitro* data suggested that TGFβ1 signaling via ALK5 was central to the injury induced astrocyte generation from SVZ glial progenitors, we asked whether antagonizing this receptor *in vivo* would decrease the extent of astrogliosis after neonatal H/I. To test this hypothesis *in vivo*, we used a hydrophilic ALK5 antagonist, SB505124 to inhibit ALK5 signaling. Studies have shown that astrocyte proliferation increases around 3–4 days of recovery [76], therefore we used this short recovery time point for studying astrogliosis within the SVZ. At 4 days of recovery from H/I we observed a significant increase in the GFAP staining intensity in the ILH SVZ vs. the CLH SVZ. This increase was significantly decreased when SB505124 was administered. Whereas the most parsimonious explanation for this result is that inhibiting ALK-5 antagonized the aberrant production of astrocytes we cannot exclude the alternative explanation that this antagonist delayed the maturation of newly generated astrocytes but did not prevent their formation. However, the injury-induced increase in GFAP expression in the neocortex appeared to be unaffected by ALK-5 inhibition. These results suggest that the decrease in GFAP expression in the SVZ is not simply due to suppression of GFAP expression. In characterizing the consequences of ALK-5 inhibition, it will be of interest to determine whether astrogliogenesis is indeed inhibited by employing markers of immature astrocytes such as vimentin or Zebrin II. These data suggest that inhibiting TGFß1-signaling through the ALK5 receptor in the early stages of cell fate determination could create an environment in the injured brain that would enable regeneration to occur more robustly. It is quite likely that by dampening the astrogliosis after injury, ALK5 inhibition may promote oligodendrogliogenesis. However, our objective for this experiment was to examine the astrocyte response after SB505124 administration. An analysis of oligodendrogliogenesis will require evaluation at a later time point after injury because the precursors must proliferate after the injury and also differentiate into fully mature oligodendrocytes. Future studies will be needed to study the long-term effects of antagonizing ALK5 signaling in the SVZ towards restoring oligodendrocyte progenitor cell production after injury.

Neonatal H/I is detrimental to the developing brain due to both its pathological effects on oligodendrocyte progenitors and neurons, but it also adversely affects white matter astrocyte development as evidenced by these studies. We hypothesized that altered cytokine signaling would affect the specification and proliferation of astrocytic precursors and immature astrocytes which would contribute to the imbalanced production of glial cells from the neonatal SVZ after brain injury. The data provided in this report fully substantiate the validity of this hypothesis. As the neonatal brain is conducive for oligodendrocyte generation, our studies suggest that a new focus of neonatal research should be on evaluating whether antagonizing those signaling pathways that induce astrocyte differentiation might restore normal oligodendrogliogenesis. The knowledge obtained from these studies may well lead to interventions that can be applied to infants after an ischemic injury or other disturbances of brain development to enable the infant brain to develop more normally, thus decreasing the incidence and lifelong neurological and psychiatric handicaps that are too often sustained.

## Methods

### Ethics Statement

All research involving animals was conducted according to relevant national and international guidelines. The animal protocol for the work described in this report was approved by the New Jersey Medical School IACUC, protocol #06069, and these studies were in accordance with the National Institute of Health Guide for the Care and Use of Laboratory Animals (NIH Publications No. 80-23) revised in 1996. We further attest that all efforts were made to minimize the number of animals used and efforts were made to ensure minimal suffering.

### Neonatal Hypoxia/Ischemia

Experiments were performed in accordance with research guidelines set forth by the New Jersey Medical School and the Society for Neuroscience Policy on the use of animals in neuroscience research. Cerebral H/I was induced in P6 rats (day of birth being P0), by a permanent unilateral (right) common carotid artery cauterization under isoflurane anesthesia followed by systemic hypoxia [2], [4], [77]. The neck wound was sutured with surgical silk and the animals were returned to the dam to recover for 1.5 h. Animals were then pre-warmed in jars for 20 min in a 37°C water bath. The pups were exposed to a humidified, hypoxic atmosphere (8% O_2_/92% N_2_) at 37°C for 75 min, allowed to recover in room air, and then returned to their dams. Control animals underwent the same procedure as experimental animals but the carotid artery was not cauterized. In experiments where an antagonist to the TGFβ1 receptor was administered, animals were treated twice daily, beginning immediately after H/I, with 5 mg/kg SB505124 or vehicle alone via intraperitoneal injection. Animals were deeply anesthetized with ketamine (75 mg/kg)/xylazine (5 mg/kg), perfused with RPMI culture medium containing 6 U/ml heparin followed by 4% paraformaldehyde, pH 7.4. Brains were removed, cryoprotected, embedded in OCT, frozen and sectioned for further analysis.

### RNA Isolation, Probe Labeling and Hybridization

SVZs from untouched control, contralateral and ILHs of H/I animals at 7 days post recovery were microdissected and snap frozen at −80°C. RNA was isolated using TRIZOL (Molecular Research Center, Inc., Cincinnati, OH). cDNA probes were prepared, labeled and hybridized to nylon Superarray stem cell array as previously described, normalizing values to GAPDH [7]. Q-PCR was performed as described earlier [73].

### Gliosphere Cultures

P4 rat pups were decapitated under sterile conditions and the brains were placed into PBS with 0.6% glucose and 2 mM MgCl_2_. Incisions were made ∼2 mm from the anterior end of the brain and ∼3 mm posterior to the first cut. Blocks were transferred to fresh PBS-glucose-MgCl_2_ and the region including the dorsolateral SVZ (SVZ_DL_) was microsurgically isolated, mechanically minced and enzymatically dissociated using a 1∶4 dilution of Accutase (Innovative Cell Technologies, CA) at 37°C for 10 min. Gliospheres were cultivated by plating the cells at 1.5×10^5^ cells/mL in ProN media (Dulbecco's modified eagle's medium/F12 media containing 10 ng/ml d-biotin, 25 µg/ml insulin, 20 nM progesterone, 100 µM putrescine, 5 ng/ml selenium, 50 µg/ml apo-transferrin, 50 µg/ml gentamycin) supplemented with 30% B104 conditioned medium [78]. Spheres generated after 7–10 days in culture were dissociated in Accutase and used for experimentation. Differentiated cells were obtained by culturing the cells with/without cytokines in N2B2 media [ProN supplemented with 0.66 mg/mL BSA and 0.5% fetal bovine serum] for 72 h. SB431542 was purchased from Tocris Cookson Inc. (Ellisville, MO).

### Western Blot Analysis

Gliospheres were dissociated with 1∶4 diluted Accutase and plated onto PdL-coated 60 mm plates in differentiation media supplemented with 2% serum. After 6 h the cells were treated with 5 ng/ml of cytokines in differentiation medium. After 48 h proteins were extracted and Western blot analysis was performed for GFAP (Dako, Carpinteria, CA), aldolase C/Zebrin (gift from Dr. Richard Hawkes), and GS (Chemicon, Temecula, CA). Total STAT3, pSTAT3 tyrosine705 and pSTAT3 serine727, total Smad2/3 and phosphorylated Smad1 and Smad 2/3 were analyzed at 30 minutes of stimulation using antibodies from Cell Signaling Technologies (Danvers, MA). The blots were stripped and reprobed with anti-β-tubulin to determine equivalent loading as described previously [79].

### Immunocytochemistry

Cells were fixed briefly with cold acetone and then incubated in rabbit anti-GFAP (1/500, Dako), rinsed well and then incubated for 2 h with fluorochome conjugated secondary antibodies (1/200, Jackson Immunoresearch). BrdU immunofluorescence was performed as previously described [80]. Cells were visualized using an Olympus AX70 microscope and imaged using a Photometrics cooled charged coupled device camera (Tucson, AZ) interfaced with IP Lab scientific imaging software (Scanalytics Fairfax, VA). At least 6 random (nonadjacent) fields were counted per well under a 40× objective and a total of 2 wells analyzed per 3 independent experiments. For the SB505124 experiment, images were taken at 20× magnification on 2 nonadjacent sections from at least 4 animals per group.

Tissue sections (12 µm) were collected and immunohistochemistry was performed. For BrdU labeling experiments, BrdU (Sigma, at 50 mg/kg body weight, 10 mg/ml in 0.007 N NaOH in 0.9% NaCl) was administered intraperitoneally (i.p.) 2 days following H/I. On day 7, animals were sacrificed by intracardiac perfusion, cryoprotected, frozen and tissue sections encompassing the region of brain subserved by the middle cerebral artery were cut at 12 µm and mounted onto glass slides. Select tissue sections were incubated for 1 h at RT in 2 N HCl, extensively rinsed with borate buffer (pH 8.5), and blocked in a solution containing 10% BSA, 10% goat serum in Tris buffer for 1 h. The sections were then incubated with a rat monoclonal anti-BrdU (Accurate Chemical, NY; 1∶30) and mouse IgG anti-vimentin (V9 clone, Roche, 1∶100) overnight at 4°C. Sections were further incubated in fluorochrome-conjugated secondary antibodies (Jackson Laboratories, PA; 1∶200) for 2 h at room temperature. DAPI was used to stain all nuclei. Other sections were stained using the Rip antibody (supernatant 1∶4) and anti-GFAP (rabbit anti-GFAP 1∶500) using the same protocol as above but omitting the HCl incubation and borate buffer rinses. Images from corresponding brain regions in ipsilateral and CLHs were captured at a standardized exposure time. IP Lab was used to measure the density of staining fluorescence. The average density was calculated for control, H/I and H/I plus SB505124 treatment.

### Retrovirus Production

Replication-deficient viruses with vsv-g coats were generated from pNIT-GFP plasmid [81], [82]. pNIT-293 cells were maintained for at least two weeks prior in 0.8 mg/ml G418 in DMEM with 10% newborn calf serum. 2–3 days before transfection, cells were split onto 7 PDL-coated plates in media lacking G418. Transfection was performed when the pNIT-293 cells were 90–95% confluent. On the transfection day, vsv-g mixture was prepared with 27 mg plasmid/100 mm plate, to make a total of 0.5 ml/plate. A separate tube was prepared with a 1∶25 dilution of Lipofectamine™ 2000 Reagent (Invitrogen, Carlsbad, CA). Tubes were mixed separately, and then mixed together followed by incubation at room temperature for 30 min. The media was removed and ∼4 mL of transfection media was added to each plate. Plates were incubated for 4 h, and checked every 20 min. to redistribute media over entire plate. At the end of 4 h, transfection media was aspirated and replaced by slowly adding 10 mL of media (without G418). Two to 3 days after transfection, the supernatant was collected on ice and filtered though a 0.45 µm cellulose filter. Conical tubes containing viral supernatant were centrifuged at 19,200 rpm in Surespin 630 rotor for 2.5 h in Sorvall ultracentrifuge at 4°C. Supernatant was aspirated and pellet diluted 1/200 in cold sterile HBSS. The tube was left on ice for 2 h, or until the pellet was completely dissolved. Viral titers were determined in CFU by incubating NIH3T3 cells with serial dilutions of retrovirus. At 48 h post infection the number of GFP+ cell clusters were counted. The CFU was calculated by multiplying the number of GFP+ cell clusters by the dilution factor. DAP retrovirus was produced and titered as described previously [83].

### Retroviral Fate Mapping

Two days after H/I (P8) 2–5 mL of DAP or pNIT replication deficient retroviruses with 8 ug/mL polybrene were stereotactically injected at a rate of 0.2 µl/min targeting the lateral ventricles of anesthetized rat pups at the following coordinates: A 1.0 mm, L 1.2 mm, D 2.5 mm. At 2, 10 or 19 days after injection animals were anesthetized with ketamine (75 mg/kg)/xylazine (5 mg/kg), perfused with RPMI culture medium containing 6 U/ml heparin followed by 4% paraformaldehyde, pH 7.4. Brains were removed, cryoprotected, frozen in OCT and sectioned for further analysis. Twenty µm sections were incubated in TBS/0.3% Triton-X-100 for 30 min, blocked for 1 h, and incubated overnight with primary antibodies against GFP (chicken anti-GFP, 1∶2,500, Aves Labs, Tigard, Oregon). Sections were washed extensively with TBS/0.05% Triton-X-100 and incubated for 4 h at RT with fluorochome-conjugated secondary antibodies (Jackson Immunoresearch). After extensive rinses, the sections were counterstained with DAPI and coverslipped with gelmount. For experiments using the DAP retrovirus, staining was performed at 10 days after injection as described previously [10] on 40 µm free floating sections and cell types were characterized morphologically as astrocytes, oligodendrocytes or indeterminate. To analyze phenotypic markers, animals were perfused 19 days after pNIT injection. Twelve µm sections were incubated in TBS/0.3% Triton-X-100 for 30 min, blocked for 1 h, and incubated overnight with primary antibodies against GSTπ (rabbit anti-GSTπ, 1∶500, MBL, Santa Cruz, CA), GFAP (mouse anti-GFAP 1∶500, Cell Signaling) and GFP (chicken anti-GFP, 1∶2,500, Aves). Sections were washed extensively with TBS/0.05% Triton-X-100 and incubated for 4 h at RT with fluorochome-conjugated secondary antibodies (Jackson Immunoresearch). After extensive rinses, the sections were counterstained with DAPI and coverslipped with gelmount. For quantitative analysis, at least 6 nonadjacent sections for each group were analyzed. Images of pNIT-infected cells with immunofluorescence were taken under 63× oil magnification. All animal work was performed according to Institutional Animal Care and Use Committee (IACUC) guidelines of UMDNJ, approved protocol #0609.

### Statistical Analyses

Results from cell culture experiments were analyzed for statistical significance by ANOVA with Fisher's PLSD post-hoc analysis or using a Student's *t* test. Error bars represent SEMs unless noted otherwise. Comparisons were interpreted as significant when associated with *p*<0.05. The REST algorithm was used to evaluate the qPCR data [84].

## Supporting Information

Figure S1 Fewer SVZ cells are infected by replication deficient retroviruses in the injured hemisphere. (A) Number of retrovirally-labeled cells counted in the hypoxic sham, contralateral and ILHs at 9 days of recovery with DAP virus. (B) Distribution of labeled cells among various brain regions. For short-term analysis of retrovirus infection, 5 ul of pNIT was injected at P8 as described above and animals sacrificed 48 h later. Cryostat sections were prepared and stained for GFP. (C,D) Representative panels of pNIT-labeled cells in contralateral (C) and ipsilateral SVZs (D) 48 h after bilateral intraventricular retrovirus injection.
(0.08 MB TIF)

Figure S2 Neocortical Astrogliosis is not blocked as a consequence of antagonizing ALK-5. Representative images of CL and IL hemispheres from H/I animals that received vehicle or twice daily i.p. SB505124 injections for 4 days and then killed and processed for immunofluorescence. Sections were stained with antibodies against GFAP. Scale bar represents 50 µm.
(7.73 MB TIF)

Table S1 Results from the SuperArray stem cell array. mRNAs pooled from at least 6 animals at 7 days of recovery were labeled and hybridized to the Nylon membrane. Fold differences in ipsilateral vs. contralateral are indicated. Values are averages from 3 experiments with 3 independent sets of mRNAs.
(0.04 MB RTF)

## References

[1] Vannucci RC, Vannucci SJ (2005). Perinatal hypoxic-ischemic brain damage: evolution of an animal model.. Developmental Neuroscience.

[2] Rice JE, Vannucci RC, Brierley JB (1981). The influence of immaturity on hypoxic-ischemic brain damage in the rat.. Annals of Neurology.

[3] Welsh FA, Vannucci RC, Brierley JB (1982). Columnar alterations of NADH fluorescence during hypoxia-ischemia in immature rat brain.. Journal of Cerebral Blood Flow & Metabolism.

[4] Vannucci RC, Lyons DT, Vasta F (1988). Regional cerebral blood flow during hypoxia-ischemia in immature rats.. Stroke.

[5] Levison SW, Rothstein RP, Romanko MJ, Snyder MJ, Meyers RL (2001). Hypoxia/ischemia depletes the rat perinatal subventricular zone of oligodendrocyte progenitors and neural stem cells.. Dev Neurosci.

[6] Romanko MJ, Rothstein RP, Levison SW (2004). Neural Stem Cells in the Subventricular Zone Are Resilient to Hypoxia/Ischemia Whereas Progenitors Are Vulnerable.. Journal of Cerebral Blood Flow & Metabolism.

[7] Felling RJ, Snyder MJ, Romanko MJ, Rothstein RP, Ziegler AN (2006). Neural stem/progenitor cells participate in the regenerative response to perinatal hypoxia/ischemia.. Journal of Neuroscience.

[8] Ness JK, Romanko MJ, Rothstein RP, Wood TL, Levison SW (2001). Perinatal hypoxia-ischemia induces apoptotic and excitotoxic death of periventricular white matter oligodendrocyte progenitors.. Developmental Neuroscience.

[9] Plane JM, Liu R, Wang TW, Silverstein FS, Parent JM (2004). Neonatal hypoxic-ischemic injury increases forebrain subventricular zone neurogenesis in the mouse.. Neurobiol Dis.

[10] Yang Z, Covey MV, Bitel CL, Jonakait GM, Levison SW (2007). Sustained Neocortical Neurogenesis after Neonatal Hypoxic/Ischemic Injury.. Annals of Neurology.

[11] Yang Z, Levison SW (2006). Hypoxia/ischemia expands the regenerative capacity of progenitors in the perinatal subventricular zone.. Neuroscience.

[12] Ong J, Plane JM, Parent JM, Silverstein FS (2005). Hypoxic-ischemic injury stimulates subventricular zone proliferation and neurogenesis in the neonatal rat.. Pediatr Res.

[13] Zaidi AU, Bessert DA, Ong JE, Xu H, Barks JD (2004). New oligodendrocytes are generated after neonatal hypoxic-ischemic brain injury in rodents.. Glia.

[14] Levison SW, Chuang C, Abramson BJ, Goldman JE (1993). The migrational patterns and developmental fates of glial precursors in the rat subventricular zone are temporally regulated.. Development.

[15] Levison SW, Goldman JE (1993). Both oligodendrocytes and astrocytes develop from progenitors in the subventricular zone of postnatal rat forebrain.. Neuron.

[16] Levison SW, Goldman JE (1997). Multipotential and lineage restricted precursors coexist in the mammalian perinatal subventricular zone.. Journal of Neuroscience Research.

[17] Levison SW, Young GM, Druckman S, Bromberg JL (1999). The subventricular zone is a source of brain macroglia but not brain microglia.. Journal of Neurochemistry.

[18] Parnavelas JG (1999). Glial cell lineages in the rat cerebral cortex.. Experimental Neurology.

[19] Zerlin M, Milosevic A, Goldman JE (2004). Glial progenitors of the neonatal subventricular zone differentiate asynchronously, leading to spatial dispersion of glial clones and to the persistence of immature glia in the adult mammalian CNS.. Dev Biol.

[20] Brazel CY (2003). Neural stem cells are resistant to apoptosis [Doctoral Dissertation]..

[21] Back SA, Luo NL, Borenstein NS, Volpe JJ, Kinney HC (2002). Arrested oligodendrocyte lineage progression during human cerebral white matter development: dissociation between the timing of progenitor differentiation and myelinogenesis.. J Neuropathol Exp Neurol.

[22] Inder TE, Warfield SK, Wang H, Huppi PS, Volpe JJ (2005). Abnormal cerebral structure is present at term in premature infants.. Pediatrics.

[23] Alagappan D LD, Felling RJ, Balan M, Kotenko SV, Levison SW (2009). Brain injury expands the numbers of neural stem cells and progenitors in the SVZ by enhancing their responsiveness to EGF.. ASN Neuro.

[24] Dohgu S, Takata F, Yamauchi A, Nakagawa S, Egawa T (2005). Brain pericytes contribute to the induction and up-regulation of blood-brain barrier functions through transforming growth factor-beta production.. Brain Res.

[25] Ronaldson PT, Demarco KM, Sanchez-Covarrubias L, Solinsky CM, Davis TP (2009). Transforming growth factor-beta signaling alters substrate permeability and tight junction protein expression at the blood-brain barrier during inflammatory pain.. J Cereb Blood Flow Metab.

[26] Bain J (2009). Glial dysgenesis caused by aberrant growth factor signaling after neonatal hypoxia ischemia..

[27] Back S, Gan X, Li Y, Rosenberg P, Volpe J (1998). Maturation-dependant vulnerability of oligodendrocytes to oxidative stress-induced death caused by glutathione depletion.. Journal of Neuroscience.

[28] Kinney HC, Back SA (1998). Human oligodendroglial development: relationship to periventricular leukomalacia.. Seminars in Pediatric Neurology.

[29] Rivkin MJ, Flax J, Mozell R, Osathanondh R, Volpe JJ (1995). Oligodendroglial development in human fetal cerebrum.. AnnNeurol.

[30] Fern R, Moller T (2000). Rapid ischemic cell death in immature oligodendrocytes: a fatal glutamate release feedback loop.. Journal of Neuroscience.

[31] Graham EM, Sheldon RA, Flock DL, Ferriero DM, Martin LJ (2004). Neonatal mice lacking functional Fas death receptors are resistant to hypoxic-ischemic brain injury.. Neurobiol Dis.

[32] Matute C, Sanchez-Gomez M, Martinez-Millan L, Miledi R (1997). Glutamate receptor-mediated toxicity in optic nerve oligodendrocytes.. Proceedings of the National Academy of Sciences USA.

[33] Ness JK, Wood TL (2002). Insulin-like growth factor I, but not neurotrophin-3, sustains Akt activation and provides long-term protection of immature oligodendrocytes from glutamate-mediated apoptosis.. Mol Cell Neurosci.

[34] Northington FJ, Ferriero DM, Flock DL, Martin LJ (2001). Delayed neurodegeneration in neonatal rat thalamus after hypoxia-ischemia is apoptosis.. Journal of Neuroscience.

[35] Northington FJ, Ferriero DM, Martin LJ (2001). Neurodegeneration in the thalamus following neonatal hypoxia-ischemia is programmed cell death.. Developmental Neuroscience.

[36] Traystman RJ, Kirsch JR, Koehler RC (1991). Oxygen radical mechanisms of brain injury following ischemia and reperfusion.. J Appl Physiol.

[37] Billiards SS, Haynes RL, Folkerth RD, Borenstein NS, Trachtenberg FL (2008). Myelin abnormalities without oligodendrocyte loss in periventricular leukomalacia.. Brain Pathol.

[38] Segovia KN, McClure M, Moravec M, Luo NL, Wan Y (2008). Arrested oligodendrocyte lineage maturation in chronic perinatal white matter injury.. Ann Neurol.

[39] Back SA, Tuohy TM, Chen H, Wallingford N, Craig A (2005). Hyaluronan accumulates in demyelinated lesions and inhibits oligodendrocyte progenitor maturation.. Nat Med.

[40] Yang Z, Levison SW (2007). Perinatal hypoxic/ischemic brain injury induces persistent production of striatal neurons from subventricular zone progenitors.. Developmental Neuroscience.

[41] Burtrum D, Silverstein FS (1994). Hypoxic-ischemic brain injury stimulates glial fibrillary acidic protein mRNA and protein expression in neonatal rats.. Experimental Neurology.

[42] Maeda Y, Matsumoto M, Hori O, Kuwabara K, Ogawa S (1994). Hypoxia/reoxygenation-mediated induction of astrocyte interleukin 6: a paracrine mechanism potentially enhancing neuron survival.. Journal of Experimental Medicine.

[43] Duggal N, Schmidt-Kastner R, Hakim AM (1997). Nestin expression in reactive astrocytes following focal cerebral ischemia in rats.. Brain Res.

[44] Saliba E, Henrot A (2001). Inflammatory mediators and neonatal brain damage.. Biol Neonate.

[45] Stoll G, Jander S, Schroeter M (1998). Inflammation and glial responses in ischemic brain lesions.. Prog Neurobiol.

[46] Chen Y, Swanson RA (2003). Astrocytes and brain injury.. J Cereb Blood Flow Metab.

[47] Swanson RA, Ying W, Kauppinen TM (2004). Astrocyte influences on ischemic neuronal death.. Curr Mol Med.

[48] Nedergaard M, Dirnagl U (2005). Role of glial cells in cerebral ischemia.. Glia.

[49] Roessmann U, Gambetti P (1986). Pathological reaction of astrocytes in perinatal brain injury. Immunohistochemical study.. Acta Neuropathol (Berl).

[50] Romanko MJ, Rothstein RP, Vannucci SJ, Meyers RL, Levison SW (2000). Stem/progenitor cells in the rat subependymal zone are vulnerable to hypoxia/ischemia.. Soc Neurosci Abst.

[51] Levison SW, Romanko MJ, Rothstein RP, Snyder MJ (2002). Hypoxia-Ischemia eliminates progenitors, but not stem cells from the perinatal subventricular zone: Consequences for brain development.. Dev Neurosci.

[52] Brazel CY, Rosti RT, Boyce S, Rothstein RP, Levison SW (2004). Perinatal hypoxia/ischemia damages and depletes progenitors from the mouse subventricular zone.. Dev Neurosci.

[53] Gressens P, Richelme C, Kadhim HJ, Gadisseux JF, Evrard P (1992). The germinative zone produces the most cortical astrocytes after neuronal migration in the developing mammalian brain.. Biol Neonate.

[54] Sizonenko SV, Camm EJ, Dayer A, Kiss JZ (2008). Glial responses to neonatal hypoxic-ischemic injury in the rat cerebral cortex.. Int J Dev Neurosci.

[55] Chang A, Tourtellotte WW, Rudick R, Trapp BD (2002). Premyelinating oligodendrocytes in chronic lesions of multiple sclerosis.. New England Journal of Medicine.

[56] Levison SW, Rothstein RP, Romanko MJ, Snyder MJ, Meyers RL (2001). Hypoxic/Ischemia depletes the perinatal subventricular zone of oligodendrocyte progenitors and neural stem cells.. Developmental Neuroscience.

[57] Sun YE, Martinowich K, Ge W (2003). Making and repairing the mammalian brain–signaling toward neurogenesis and gliogenesis.. Semin Cell Dev Biol.

[58] Burrows RC, Wancio D, Levitt P, Lillien L (1997). Response diversity and the timing of progenitor cell maturation are regulated by developmental changes in EGFR expression in the cortex.. Neuron.

[59] Viti J, Feathers A, Phillips J, Lillien L (2003). Epidermal growth factor receptors control competence to interpret leukemia inhibitory factor as an astrocyte inducer in developing cortex.. Journal of Neuroscience.

[60] Molne M, Studer L, Tabar V, Ting YT, Eiden MV (2000). Early cortical precursors do not undergo LIF-mediated astrocytic differentiation.. Journal of Neuroscience Research.

[61] Johe KK, Hazel TG, Muller T, Dugich-Djordjevic MM, McKay RDG (1996). Single factors direct the differentiation of stem cells from the fetal and adult central nervous system.. Genes and Development.

[62] Richards LJ, Kilpatrick TJ, Dutton R, Tan SS, Gearing DP (1996). Leukaemia inhibitory factor or related factors promote the differentiation of neuronal and astrocytic precursors within the developing murine spinal cord.. European Journal of Neuroscience.

[63] D'Alessandro JS, Yetz-Aldape J, Wang EA (1994). Bone morphogenetic proteins induce differentiation in astrocyte lineage cells.. GrowthFactors.

[64] Mehler MF, Mabie PC, Zhu G, Gokhan S, Kessler JA (2000). Developmental changes in progenitor cell responsiveness to bone morphogenetic proteins differentially modulate progressive CNS lineage fate.. Developmental Neuroscience.

[65] Loo DT, Althoen MC, Cotman CW (1994). Down regulation of nestin by TGF-beta or serum in SFME cells accompanies differentiation into astrocytes.. Neuroreport.

[66] Lazar LM, Blum M (1992). Regional distribution and developmental expression of epidermal growth factor and transforming growth factor-alpha mRNA in mouse brain by a quantitative nuclease protection assay.. J Neurosci.

[67] Chao CC, Hu S, Tsang M, Weatherbee J, Molitor TW (1992). Effects of transforming growth factor-beta on murine astrocyte glutamine synthetase activity. Implications in neuronal injury.. J Clin Invest.

[68] Agnihotri S, Wolf A, Picard D, Hawkins C, Guha A (2009). GATA4 is a regulator of astrocyte cell proliferation and apoptosis in the human and murine central nervous system.. Oncogene.

[69] Hunter KE, Sporn MB, Davies AM (1993). Transforming growth factor-betas inhibit mitogen-stimulated proliferation of astrocytes.. Glia.

[70] Robe PA, Rogister B, Merville MP, Bours V (2000). Growth regulation of astrocytes and C6 cells by TGFbeta1: correlation with gap junctions.. Neuroreport.

[71] Woodruff RH, Franklin RJ (1999). Demyelination and remyelination of the caudal cerebellar peduncle of adult rats following stereotaxic injections of lysolecithin, ethidium bromide, and complement/anti-galactocerebroside: a comparative study.. Glia.

[72] Nakashima K, Yanagisawa M, Arakawa H, Taga T (1999). Astrocyte differentiation mediated by LIF in cooperation with BMP2.. FEBS Letters.

[73] Covey MV, Levison SW (2007). Leukemia inhibitory factor participates in the expansion of neural stem/progenitors after perinatal hypoxia/ischemia.. Neuroscience.

[74] Inman GJ, Nicolas FJ, Callahan JF, Harling JD, Gaster LM (2002). SB-431542 is a potent and specific inhibitor of transforming growth factor-beta superfamily type I activin receptor-like kinase (ALK) receptors ALK4, ALK5, and ALK7.. Mol Pharmacol.

[75] Laping NJ, Grygielko E, Mathur A, Butter S, Bomberger J (2002). Inhibition of transforming growth factor (TGF)-beta1-induced extracellular matrix with a novel inhibitor of the TGF-beta type I receptor kinase activity: SB-431542.. Mol Pharmacol.

[76] Buffo A, Rite I, Tripathi P, Lepier A, Colak D (2008). Origin and progeny of reactive gliosis: A source of multipotent cells in the injured brain.. Proc Natl Acad Sci U S A.

[77] Vannucci RC, Connor JR, Mauger DT, Palmer C, Smith MB (1999). Rat model of perinatal hypoxic-ischemic brain damage.. J Neurosci Res.

[78] Young G, Levison S (1997). An improved method for propagating oligodendrocyte progenitors in vitro.. Journal of Neuroscience Methods.

[79] Basu A, Krady JK, O'Malley M, Styren SD, DeKosky ST (2002). The type 1 interleukin-1 receptor is essential for the efficient activation of microglia and the induction of multiple proinflammatory mediators in response to brain injury.. J Neurosci.

[80] Young GM, Albrecht PJ, Levison SW (1996). In vitro differentiation of neurons and astrocytes from juvenile SVZ cells: Rescue of progenitors fated to die?. Soc Neurosci Abst.

[81] Milosevic A, Goldman JE (2002). Progenitors in the postnatal cerebellar white matter are antigenically heterogeneous.. J Comp Neurol.

[82] Kakita A, Goldman JE (1999). Patterns and dynamics of SVZ cell migration in the postnatal forebrain: monitoring living progenitors in slice preparations.. Neuron.

[83] Levison SW, Goldman JE (1993). Both oligodendrocytes and astrocytes develop from progenitors in the subventricular zone of postnatal rat forebrain.. Neuron.

[84] Pfaffl MW, Horgan GW, Dempfle L (2002). Relative expression software tool (REST) for group-wise comparison and statistical analysis of relative expression results in real-time PCR.. Nucleic Acids Res.

