# Supplementary figures and images for: TGFß1 Stimulates the Over-Production of White Matter Astrocytes from Precursors of the “Brain Marrow” in a Rodent Model of Neonatal Encephalopathy
Source: PLoS One. 2010 Mar 5;5(3):e9567. doi: 10.1371/journal.pone.0009567 (PMC2832687; doi:10.1371/journal.pone.0009567)

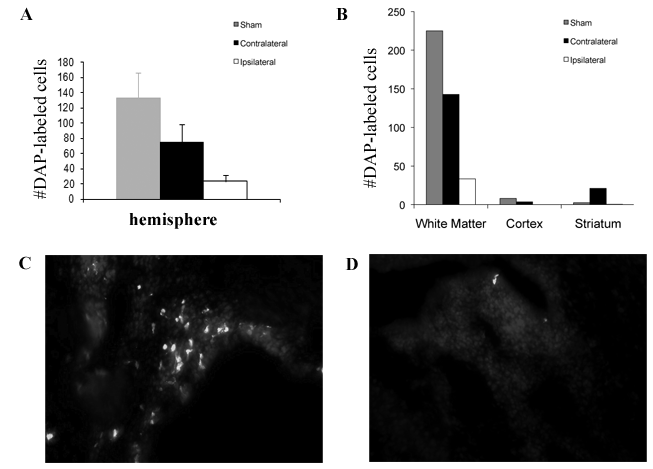

Supplement: Figure S1 — Fewer SVZ cells are infected by replication deficient retroviruses in the injured hemisphere. (A) Number of retrovirally-labeled cells counted in the hypoxic sham, contralateral and ILHs at 9 days of recovery with DAP virus. (B) Distribution of labeled cells among various brain regions. For short-term analysis of retrovirus infection, 5 ul of pNIT was injected at P8 as described above and animals sacrificed 48 h later. Cryostat sections were prepared and stained for GFP. (C,D) Representative panels of pNIT-labeled cells in contralateral (C) and ipsilateral SVZs (D) 48 h after bilateral intraventricular retrovirus injection. (0.08 MB TIF) [file pone.0009567.s001.tif]

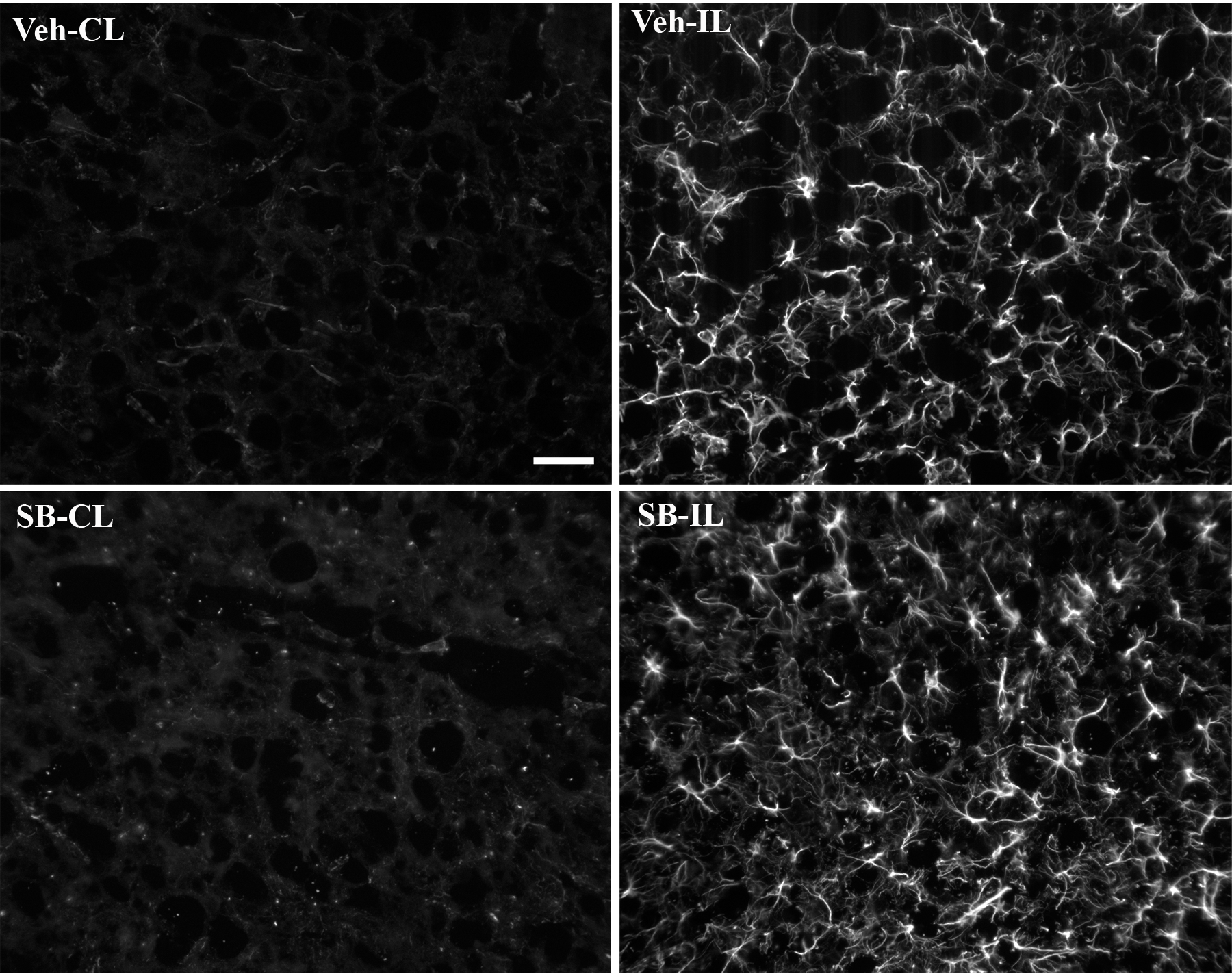

Supplement: Figure S2 — Neocortical Astrogliosis is not blocked as a consequence of antagonizing ALK-5. Representative images of CL and IL hemispheres from H/I animals that received vehicle or twice daily i.p. SB505124 injections for 4 days and then killed and processed for immunofluorescence. Sections were stained with antibodies against GFAP. Scale bar represents 50 µm. (7.73 MB TIF) [file pone.0009567.s002.tif]
