# Supplementary material for: TGFß1 Stimulates the Over-Production of White Matter Astrocytes from Precursors of the “Brain Marrow” in a Rodent Model of Neonatal Encephalopathy
Source: PLoS One. 2010 Mar 5;5(3):e9567. doi: 10.1371/journal.pone.0009567 (PMC2832687; doi:10.1371/journal.pone.0009567)
Supplement: Table S1 — Results from the SuperArray stem cell array. mRNAs pooled from at least 6 animals at 7 days of recovery were labeled and hybridized to the Nylon membrane. Fold differences in ipsilateral vs. contralateral are indicated. Values are averages from 3 experiments with 3 independent sets of mRNAs. (0.04 MB RTF) [file pone.0009567.s003.rtf]

Table S1.  Results from the SuperArray stem cell array

Family and its receptors	Gene	Fold change at 7 days of recovery	
Fibroblast Growth Factor (FGF) & receptors 	FGF2
FGF4
FGF10
FGF17
FGFR2
FGFR3	2.5
8.92
2.67
2.73
0.43
0.45	
Epidermal Growth Factor (EGF) 	EGF
EGFR 	4.34
0.98	
Transforming growth factor b (TGFb) & receptor 


Bone morphogenetic proteins (BMPs) & receptor	TGFb1
ALK1
ALK5
BMP2
BMP10
BMPR2	4.1
2.30
1.96
0.53
0.54
0.422	
Interleukin 6 (IL6) & receptor 	IL6
IL6 Ra
LIF
LIFR
Gp130	0.162
4.84
1.02
1.26
1.47	
Notch 	Notch1
Notch3	0.099
2.22	
Markers	S100b
Vimentin
CD44	2.2
3.1
1.8	
